# Serum lactate in anhepatic patients and the impact of continuous renal replacement therapy on its clearance: a case series

**DOI:** 10.1016/j.bjane.2024.844542

**Published:** 2024-07-19

**Authors:** Rodolpho Augusto de Moura Pedro, Paula Sepulveda Mesquita, Frederico Almeida Baptista de Oliveira Filho, Bruna Carla Scharanch, Luís Augusto Carneiro D'Albuquerque, Luís Marcelo Sá Malbouisson

**Affiliations:** Hospital das Clínicas da Faculdade de Medicina de São Paulo, Unidade de Terapia Intensiva de Fígado e Gastroenterologia, São Paulo, SP, Brazil

Dear Editor,

Serum lactate is a well-recognized marker of hypoperfusion^1^ as its production increases during the anaerobic metabolism of tissue hypoxia. However, elevated lactate levels can also occur under non-hypoxic conditions due to accelerated glycolysis (e.g., in hyperthyroidism, hyper catabolism, adrenaline, salbutamol), congenital disorders, thiamine deficiency, alkalemia, lactate infusion, or under impaired liver metabolism.^2^

Reduced clearance can partially explain lactate levels during acute liver failure or anhepatic status (e.g., transplant anhepatic phase or full hepatectomy as rescue therapy for a malfunctioning “toxic graft”). This metabolic deficiency might be mitigated by enhanced removal through Continuous Renal Replacement Therapy (CRRT).^3^ Therefore, lactate levels in anhepatic status can be misleading, as high values may not represent hypoperfusion, and decreases under CRRT may not translate clinical improvement.

The literature on lactate dynamics during anhepatic status is limited, and there is even less data on the impact of CRRT in this phase. Our main objective in this case series is to describe the evolution of serum lactate during liver absence and the impact of CRRT on its clearance. We present three cases of patients maintained anhepatic after full rescue hepatectomy with a porto-caval shunt until a new liver was available for transplantation.

From February 1, 2020, to February 1, 2021, all patients admitted to the liver critical unit at the Hospital das Clínicas – Universidade de São Paulo, who underwent a full hepatectomy with porto-caval shunt before a new organ was available for transplantation were retrospectively screened. Informed consent was obtained from the patients or their legal representatives. Serum lactate levels were retrieved if collected from an arterial or central venous source. Given the rarity of these events, a convenience sample size was defined with all eligible cases. This manuscript adheres to the CARE guideline for case reports/case series and was approved by the research and ethics Committee (06/16/2023, approval number 6.122.526).

Three patients met the inclusion criteria. In all cases, the responsible parties provided written informed consent for inclusion in the following description. The relationship between serum lactate, anhepatic status and CRRT over time is presented in Figure 1, and cases are described as follows:

**Figure 1:**
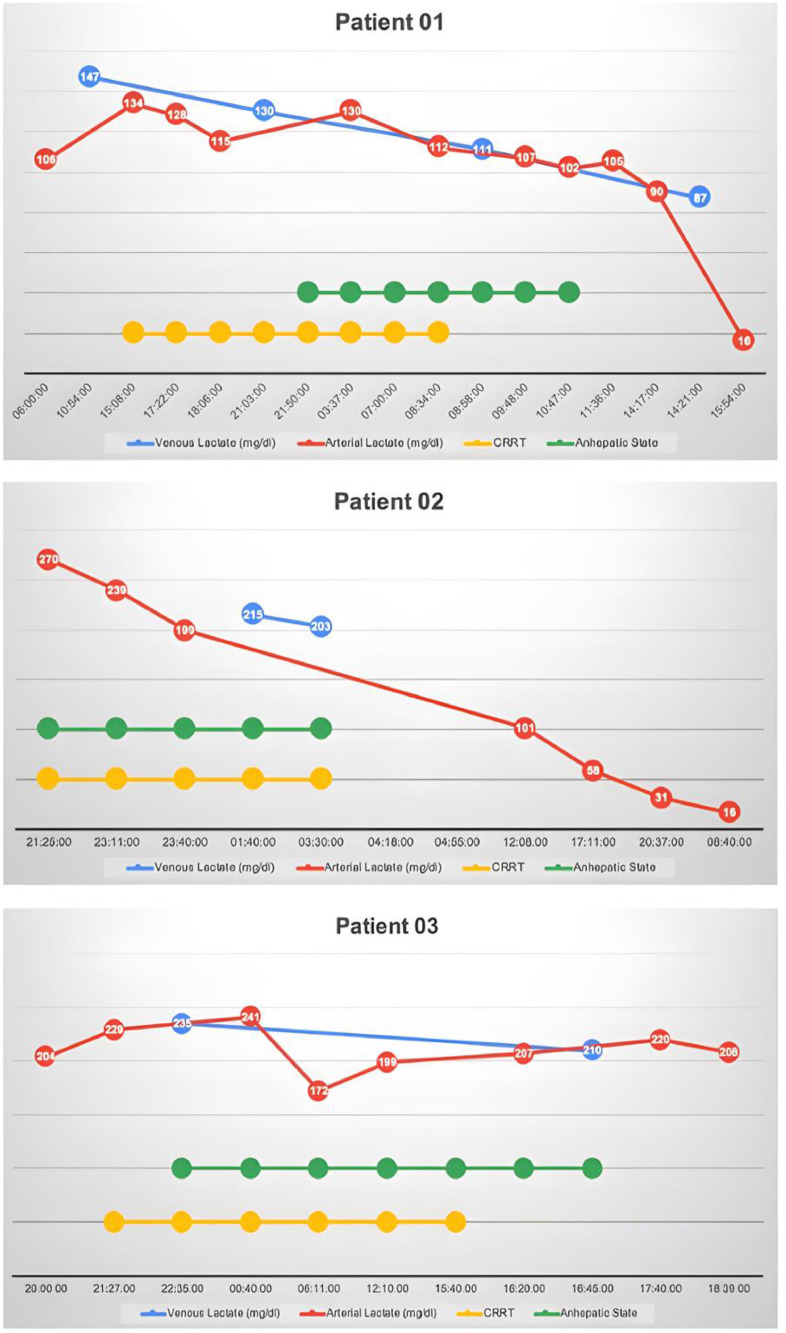
Serum lactate correlation with CRRT in patients 1, 2 and 3.

A 50-year-old male developed shock and hyperlactatemia on the first postoperative day after liver transplant. On the second postoperative day, he experienced worsening hemodynamics, liver function tests and hepatic encephalopathy. Diagnosed with liver graft primary nonfunction, CRRT with hemofiltration was initiated, and he was listed for a new transplant. Due to clinical deterioration, a rescue full hepatectomy with a porto-caval shunt anastomosis was performed. The patient remained anhepatic for 12 hours and 57 minutes until the new organ was transplanted. During this period, CRRT was maintained, resulting in decreased lactate levels even before transplantation. A more significant decrease in lactate levels occurred after the new liver was implanted. The patient recovered well after surgery and was discharged home a few weeks later.

A 37-year-old female with acute liver failure due to sulfasalazine was listed for liver transplant. While awaiting a new organ, she experienced progressive shock and worsening hepatic encephalopathy, leading to CRRT initiation. After a donor became available, a rescue full hepatectomy with porto-caval shunt was performed. The patient remained anhepatic in the operating room for 6 hours and 2 minutes until the organ was transplanted. Serum lactate levels started to decrease after CRRT initiation and fell progressively during the anhepatic phase. After the liver transplant, hemodynamics improved, but the patient developed bilateral mydriasis followed by confirmation of cerebral death.

A 63-year-old female developed refractory shock and hyperlactatemia immediately after a new liver was transplanted. Due to rapid hemodynamic deterioration, she was relisted and prioritized for a new organ. CRRT was initiated and a rescue full hepatectomy with porto-caval shunt was performed.The patient remained anhepatic for 18 hours and 10 minutes. Initially, serum lactate decreased with CRRT implementation. Despite the new liver transplant, there was no improvement in hemodynamics; lactate levels increased again, and the patient died hours later.

In this case series, we present three instances of prolonged anhepatic status with hyperlactatemia, emphasizing that elevated lactate levels can result from the absence of liver metabolism and that these levels can decrease under CRRT even before a new liver is implanted. Severe liver dysfunction may contribute to clinical deterioration due to the release of cytokine and vasodilatory substances, causing inflammation, acidosis and shock.^4^ In this scenario, removing the “toxic liver” is described as rescue therapy to contain the cytokine storm, stabilize hemodynamics, and gain time for a new liver transplant.^5^ This decision is complex, as the removal of the old liver initiates a critical waiting period. CRRT is commonly used in this context to prevent hyperammonemia and hopefully protect the brain, as the absence of liver simulates acute liver failure.

Previous literature indicates that lactate clearance is also impaired in this context,^5^ reinforcing the concept that lactate levels should not be used exclusively as a surrogate of hypoperfusion. The lactate removal after CRRT initiation, even in the complete absence of liver, supports the hypothesis of external clearance and should not be interpreted as hemodynamic improvement. Although this case series represents a small single-center experience, we believe these data contribute to a better understanding of how lactate can be misleading in severe liver dysfunction or liver absence and how CRRT impacts its clearance.

Hyperlactatemia during anhepatic status decreased after CRRT initiation. Therefore, lactate levels should not be directly interpreted as a surrogate for tissue perfusion in this scenario.

## Conflicts of interest

The authors declare no conflicts of interest.
